# Modeling effects of intrinsic and extrinsic rewards on the competition between striatal learning systems

**DOI:** 10.3389/fpsyg.2013.00739

**Published:** 2013-10-16

**Authors:** Joschka Boedecker, Thomas Lampe, Martin Riedmiller

**Affiliations:** Machine Learning Lab, Department of Computer Science, Faculty of Engineering, Albert-Ludwigs-University FreiburgFreiburg, Germany

**Keywords:** striatal models, reinforcement learning model, model-free vs. model-based learning, intrinsic motivation, extrinsic motivation

## Abstract

A common assumption in psychology, economics, and other fields holds that higher performance will result if extrinsic rewards (such as money) are offered as an incentive. While this principle seems to work well for tasks that require the execution of the same sequence of steps over and over, with little uncertainty about the process, in other cases, especially where creative problem solving is required due to the difficulty in finding the optimal sequence of actions, external rewards can actually be detrimental to task performance. Furthermore, they have the potential to undermine intrinsic motivation to do an otherwise interesting activity. In this work, we extend a computational model of the dorsomedial and dorsolateral striatal reinforcement learning systems to account for the effects of extrinsic and intrinsic rewards. The model assumes that the brain employs both a goal-directed and a habitual learning system, and competition between both is based on the trade-off between the cost of the reasoning process and value of information. The goal-directed system elicits internal rewards when its models of the environment improve, while the habitual system, being model-free, does not. Our results account for the phenomena that initial extrinsic reward leads to reduced activity after extinction compared to the case without any initial extrinsic rewards, and that performance in complex task settings drops when higher external rewards are promised. We also test the hypothesis that external rewards bias the competition in favor of the computationally efficient, but cruder and less flexible habitual system, which can negatively influence intrinsic motivation and task performance in the class of tasks we consider.

## 1. Introduction

What motivates intelligent beings to perform certain actions in their environment is a central question in psychology. The influential paradigm of operant conditioning by Skinner (1953) held that all behavior is stimulated by external rewards presented to an animal. This view was challenged, however, by observations made by White (1959) that some behaviors are *intrinsically motivated*, i.e., they are performed simply because the activity is *intrinsically rewarding*. Deci (1971) then examined what effects external rewards would have on intrinsic motivation and found that under certain circumstances, extrinsic rewards could undermine intrinsic motivation. Later on, several studies (see extensive meta-analytic review by Deci et al., 1999) observed that external rewards can decrease cognitive flexibility in problem solving (McGraw and McCullers, 1979), and have the potential to decrease performance on complex tasks (Erez et al., 1990). These findings significantly contradicted predictions of earlier theories such as operant conditioning or utility theory in economics.

To explain these observations, several theoretical accounts have been put forward [e.g., Cognitive Evaluation Theory by Deci and Ryan (1985), Attribution Theory by Lepper et al. (1973), or Self-Determination Theory by Ryan and Deci. (2000) amongst others] which suggest different cognitive mechanisms to account for the data. However, it is not clear what *computational* mechanisms in the brain could give rise to these phenomena. A computational model would enable quantitative comparisons of different hypotheses, test various experimental settings, and generate predictions for new, untested scenarios.

Here, we provide such a computational model by extending two previously presented models explaining behavioral control in the decision systems (Daw et al., 2005), and trade-offs between habitual and goal-directed brain processes (Keramati et al., 2011). Both of these models follow a hypothesis from behavioral economics, suggesting that two distinct control systems in the brain compete for control of actions (see e.g., Kahneman and Frederick, 2002). The models are formalized using the framework of reinforcement learning (RL, see e.g., Sutton and Barto, 1998), and it is assumed that one controller uses computationally efficient model-free RL, whereas the other one uses statistically efficient model-based RL algorithms. The model-free system represents a habitual process, implementing a cache of efficient actions for a given situation, while the model-based system realizes a goal-directed process by searching a tree of recorded state-action transition probabilities for alternative choices. Both computational models could account for several phenomena from animal experiments designed to test devaluation resistance, including habituation after extensive training, non-habituation in ambivalent tasks, and habituation in preference tasks. Our proposed model is a mixture of both earlier models (see below for details), and, for the first time, connects them to intrinsic rewards for the model-based goal-directed subsystem. With this extension, we aim to explain three additional phenomena which the previous models could not account for.

### 1.1. Activity without extrinsic reward

When dealing with a creative or complex system, both humans and animals can be observed to interact (to “play”) with it even if no extrinsic reward whatsoever is being provided or promised.

### 1.2. Reduced post-extinction activity

In creative tasks, the presence of strong extrinsic rewards can lead to diminished activity after said rewards have been devalued. More specifically, the activity will be lower than it would have been had the subject never received any extrinsic reward in the first place (Deci, 1971). Strong extrinsic rewards are therefore expected to suppress intrinsic motivation.

### 1.3. Effects of promised external rewards

It has been observed that the promise of strong extrinsic rewards for a certain level of task performance does not only lead to diminished activity during creative problem solving as described above, but in fact also leads to inferior final performance on tasks involving cognitive skills (Ariely et al., 2009).

## 2. Materials and methods

Since our model is an extension of the work by Daw et al. (2005) and Keramati et al. (2011) on striatal competition, we first give a brief description of their respective approaches. After that, we will detail the changes that were newly introduced in detail.

### 2.1. Striatal competition

Both previous models intend to give a formal account of the decision system and its division into a goal-directed and a habitual module. The former realizes a model-based “tree” system that gradually builds a comprehensive model of the task, which can then be used to find an optimal sequence of steps that results in the greatest reward for a given task.

In contrast, the habitual system learns in a model-free fashion as a “cache,” retaining only the knowledge of which possible action in a given situation promises a higher final payoff, but does not record which subsequent state the action would lead to. This makes it computationally cheaper than the goal-directed system, but also less adaptive to changes in the environment.

In Daw et al. (2005), these systems are assumed to be located in the prefrontal cortex and the dorsolateral striatum, respectively. While newer studies have placed the goal-directed system in the dorsomedial striatum (Yin et al., 2005), the functional distinction between the two types of system remains unchallenged.

Reproducing these aspects in the models allows them to explain several observations regarding habituation in animals. Specifically, it was found by Killcross and Coutureau (2003) as well as Holland (2004) that if a rat performs a simple lever-pulling task long enough that generates a food reward, it will become resistant to devaluation. Even if the food reward is being negated (via poison), the animal will continue performing the same sequence of actions. If the devaluation occurs after only moderate training, no such resistance occurs, and the rat will immediately adapt its behavior.

It was argued that the observed effects are caused by the competition between both modules. The adaptable goal-directed system is active initially, but replaced by the habitual system after extended training, at which point the agent becomes resistant to devaluation. The main difference between the two models lies in the specific competition mechanism used to arbitrate between both systems.

#### 2.1.1. Uncertainty-based competition

In the earlier model by Daw et al. (2005), it is assumed that the system is chosen which is more certain about the action to be taken. To determine uncertainty, both the model-based and the model-free system are implemented using Bayesian Reinforcement Learning Dearden et al., 1998; Mannor et al., 2004. Therefore, rather than learning *Q*-values for a given state, they assume a prior (Beta) distribution over *Q*-values for each entry in the *Q*-table. Bayesian updates are then used to calculate the posterior distribution based on the experience during learning. Likewise, the transition function and the terminal reward function employed by the model-based subsystem are also tables of distributions. A policy is then generated through tree-search on this model, which is realized by performing Value Iteration on a *Q*-function initialized to the reward function.

When the system enters some state *s*, the value distribution *Q_s, a_* is determined for each available action *a*. For each *a*, either the goal-directed or the habitual system's estimate of *Q_s, a_* is used. The system that provides the *Q*-distribution is chosen depending on which one has the lower variance σ^2^:

(1) $$Q_{s,\;a}^{*}=\{\begin{array}{cc} Q_{s,\;a}^{tree} & \text{if}{\left(\sigma_{s,\;a}^{2}\right)}^{\text{tree}}\\ Q_{s,\;a}^{cache} & \text{otherwise} \end{array}\begin{array}{c} <{\left(\sigma_{s,\;a}^{2}\right)}^{\text{cache}} \end{array}$$

After selecting the more confident system for each action, the actual action to be performed is chosen through Boltzmann exploration over the *Q*-distributions' means μ^*^, parameterized by the softmax parameter β.

(2) $$P\left(a=a_{i}|s\right)\propto e^{{\beta \mu }_{s,\;a_{i}}^{*}}$$

At each time step, all distribution parameters decay exponentially with a forgetting factor θ to their priors, thus keeping the system capable of learning from new experiences even after long training durations.

Since the tree-search is performed until convergence at each time step, a sudden change in the reward model resulting from a devaluation event will immediately be propagated all the way through the state space. In contrast, the model-free system will have to perform the original sequence several times to register a change in the terminal state's value in the starting state. The habitual system becomes dominant after extended training, but not after moderate one, since its variance decreases more slowly than that of the goal-directed system. Thus, the model accounts for the empirical findings.

#### 2.1.2 Value-based competition

Keramati et al. (2011) modify the basic approach of Daw et al. (2005) by using the value of perfect information (VPI) instead of uncertainty. Here, the model-free system computes how much value would be gained from knowing the true value of a given action. Such knowledge would only have value if it allows the agent to improve its policy. Therefore, it should reveal that the previously preferred action is not in fact optimal, either by showing that its true value is less than thought, or that another action promises higher rewards. Formally, the gain *G* of knowing that an action *a* has the value *Q_s, a_* = *x* can be computed as follows, where the calculation differs depending on whether *a* is the optimal action *a*_1_ or second best action *a*_2_ as judged by the habitual system thus far.

(3) $$G_{s,\;a}\left(x\right)=\{\begin{array}{cc} Q_{s,\;a_{2}}^{\text{cache}}-x & \text{if }a=a_{1}\text{ and}\\ & x<Q_{s,\;a_{2}}^{\text{cache}}\;\\ x-Q_{s,\;a_{1}}^{\text{cache}} & \text{if }a\neq \;a_{1}\text{ and}\\ & x>Q_{s,\;a_{1}}^{\text{cache}}\;\\ 0 & \text{otherwise} \end{array}$$

The VPI is then simply given by the expected Gain over the distribution of possible values that *Q_s, a_* can take.

(4) $$\text{VPI}\left(s,\;a\right)=E\left[G_{s,\;a}\left(x\right)\right]$$

Intuitively and generally speaking, this value is higher if an action's *Q*-distribution overlaps strongly with the best action, since in this case the former may turn out to be preferable. Conversely, once the distributions have separated, knowing the true value of an action is unlikely to change which one is ultimately chosen.

Once computed, the VPI is compared against the costs of opportunity for performing a tree-search, denoted by *R*τ, with *R* being the expected average reward and τ being the cost in terms of deliberation time for traversing an edge of the tree.

(5) $$Q_{s,\;a}^{*}=\{\begin{array}{cc} Q_{s,\;a}^{\text{tree}} & \text{if VPI}\left(s,\;a\right)>\bar{R}\tau \\ Q_{s,\;a}^{\text{cache}} & \text{otherwise } \end{array}$$

Only if the VPI is higher than the opportunity costs is the model-based system activated to determine the true reward. The winning system's estimate is then used for action selection. Since determining the VPI does not involve the goal-directed system in any way, this approach better adheres to the assumption that using the habitual system is less time-intensive.

Finally, the average reward *R* is updated with new observations *r* using learning rate η:

(6) $${\bar{R}}_{t+1}=\left(1-\eta \right){\bar{R}}_{t}+\eta r_{t}$$

One advantage of using the VPI instead of both modules' uncertainty lies primarily in considerations of speed. Since the VPI can be computed purely from the habitual system's uncertainty about the value distribution, thus often eliminating the need for the costly computations required when activating the goal-directed system. In contrast, the previous model always required the calculation of the goal-directed system's uncertainty and value. Without the ability to speed up the decision process, that would raise the issue of why a habitual module should even have evolved.

It is worth noting that the goal-directed system used both here and by Keramati et al. (2011) does not initially provide perfect value estimates, making the term “value of perfect information” somewhat incorrect. As such, it may not fulfill its purpose of improving the action choices at the very beginning of the learning process. However, its ability to reason globally allows it to learn sensible actions from fewer observations than the rigid cache, and thus to provide value estimates soon.

### 2.2. Model extension

Aside from using a mixture of the features present in our predecessor models, there are two major extensions in our model that were not present in its predecessors, which will be described in detail in the following.

#### 2.2.1. Intrinsic rewards

The main contribution of our model lies in its extension with a mechanism for intrinsic motivation. The central feature of intrinsic rewards lies in that their value depends on the current state of the model, as opposed to extrinsic rewards that are provided by the process or environment. As such, intrinsic rewards can notably arise *only* in the goal-directed system, and are not applied to the habitual one.

Currently we consider only one of multiple types of intrinsic reward, namely the learning progress of the transition model (similar to Oudeyer et al., 2007). Learning progress is based on the intuition that a system should explore regions where it can currently learn the most based on the state of its internal models, i.e., make the largest progress at improving its models. In contrast, simple metrics based on surprise are prone to get stuck in completely unpredictable situations which is avoided by rewarding progress (i.e., reduction of surprise over time) instead. There are other proposed aspects to intrinsic motivation, such as competence-based and information-theoretical mechanisms (for an overview, see section 4.1), but we focus on progress for the sake of simplicity, as it already accounts for the phenomena we consider by itself. As measure of learning progress we use the magnitude of shifts in the means of the transition function's distributions. Formally, the intrinsic reward *I* for choosing action *a* in state *s* is given by the equation:

(7) $$I_{s,\;a}=\iota \sum_{s^{\prime }\;\in \;S}\left|\Delta \mu_{s,\;a,\;s^{\prime }}^{\text{trans}}\right|$$

Here, ι is a factor used to accentuate the intrinsic rewards and bring them into the same order of magnitude as the extrinsic ones.

*I* is then added to the result of the tree-search:

(8) $${\tilde{Q}}_{s,\;a}^{\text{tree}}:=Q_{s,\;a}^{\text{tree}}+I_{s,\;a}$$

The resulting *Q*-values ${\tilde{Q}}^{\text{tree}}$ are then used in place of those determined by the search for the purpose of subsystem selection and exploration.

At this point, one may wonder why, from among the many alternative types of intrinsic motivation, we choose Δμ^trans^ rather than Δ(σ^2^)^trans^, which provides a more meaningful measure of learning progress. Our model provides the variance readily, but when using Dirichlet distributions with a large number of states, the variance is not a useful metric. This is because shifts in variance for observations that have or have not been made before differ very little. Only once the transition model is nearly stable will unexpected observations cause a distinct shift. However, by that time, the intrinsic rewards will be too low to have significant influence on action selection anyway.

#### 2.2.2. Transition costs

Aside from intrinsic rewards, we also introduce transition costs. While a common element of RL and formalized in the Bellman Equation (see Sutton and Barto, 1998), they were not present in the model by Daw et al. (2005). Instead, the entire terminal reward of a trajectory was propagated all the way to the starting state.

By accommodating them, we enable the model to acquire minimum-time policies in tasks where trajectories can contain loops. Most importantly, transition costs can also be chosen differently for each action, thereby modeling energy conservation.

It is worth noting that action-based transition costs do not fall cleanly into the distinction between extrinsic and intrinsic rewards. Traditionally considered extrinsic rewards, they are likewise applied to the habitual system, as opposed to intrinsic rewards, which due to being model-based can naturally only occur within the goal-directed system. On the other hand, they mimic intrinsic rewards in that they are essentially inherent—one may be tempted to say “intrinsic”—to the agent. Action costs are not provided by the environment, and can thus be assumed to occur even when other extrinsic rewards do not. To avoid confusion, we will dub them *action rewards* in the following and mention explicitly when they are used and when not, since their appearance is not bound to either of the two major reward types.

Applying transition costs can easily be done during both tree-search and update of the habitual system by adding them to the discounted extrinsic reward that would result from choosing the optimal action a_*_ in the successor state *s′*. Doing so yields a new target mean $\hat{\mu }$:

(9) $${\hat{\mu }}_{s,\;a}={\gamma \mu }_{s^{\prime },\;a_{*}}+r_{a}$$

The update rule for the distribution parameters also requires the second moments of the successor states' Beta distributions. We therefore generate a new distribution ${\hat{Q}}_{s,\;a}=\text{Beta}\left(\hat{\alpha },\;\hat{\beta }\right)$ with the target mean ${\hat{\mu }}_{s,\;a}$, from which we can then infer these moments. Between its parameters, the following relationship must hold:

(10) $$\frac{\hat{\alpha }}{\hat{\mu }}=\frac{\hat{\beta }}{1-\hat{\mu }}$$

**Figure 1:**
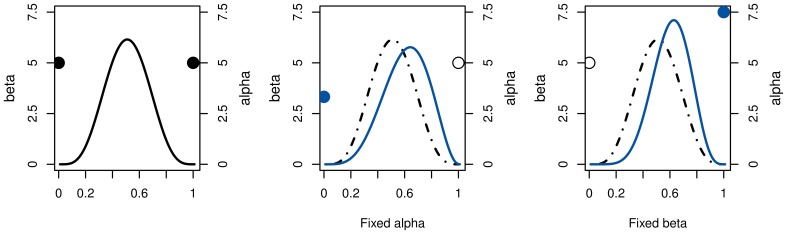
**Illustration of the relationship between Beta parameters for a given positive mean shift of an arbitrary distribution (left)**. If α is fixed (**middle**), the lower β results in a flatter distribution with higher variance. Conversely, fixing β would reduce the variance (**right**). For action costs, i.e., negative action rewards, the effects are reversed.

Thus, we need to fix one of the Beta parameters to determine the other. Depending on which one is chosen, the distribution's variance may either increase or decrease, as illustrated in Figure 1. Under the reasonable assumption that every step of tree-search introduces additional uncertainty, we choose whichever would cause a variance increase.

The resulting ${\hat{Q}}_{s,\;a}$ is then used for the computation of the new distribution parameters. Analogously to Daw et al. (2005), they are updated using a mixture rule derived from Dearden et al. (1998).

(11) $$\int_{0}^{1}\text{Beta}\left(\alpha_{s,\;a}+x,\;\beta_{s,\;a}+\left(1-x\right)\right){\hat{Q}}_{s,\;a}\left(x\right)\text{d}x$$

Details on the closed-form update can be found in the supplemental material to Daw et al. (2005).

#### 2.2.3. Model mixture

Like Keramati et al. (2011), we use the VPI to mediate between the goal-directed and the habitual subsystem. The alternative approach of using the variance of the *Q*-function's estimates would not be plausible in a framework containing intrinsic rewards. Intrinsic motivation is generally assumed to be high for regions of the state space in which the model has not been learned yet. In these regions, the goal-directed system's variance will also be particularly high (Oudeyer and Kaplan, 2007). If the goal-directed system's variance is involved in the competition mechanism, this will lead to it being rejected in precisely those situations when intrinsic motivation is high, thereby neutralizing the effect of the latter.

**Figure 2:**
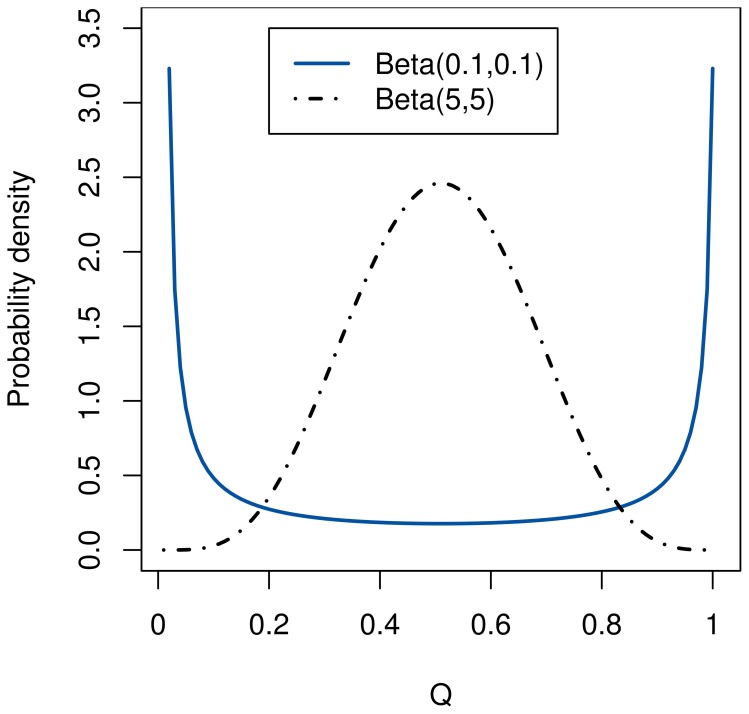
**Unlike a Gaussian, a Beta distribution can represent both double-peaked (with α, β < 1) and single-peaked (with α, β > 1) distributions**.

From the original approach by Daw et al. (2005) we retain the use of Beta and Dirichlet distributions to represent the model and the policies learned by the agent, as opposed to the Gaussians used by Keramati et al. (2011). Using a Beta distribution for the policy carries the advantage that its probability density function can have two peaks, as illustrated in Figure 2. Therefore, it is able to represent a limited amount of ambiguity arising from non-determinism, while single-peaked Gaussians model only uncertainty. Since their range is constrained in the interval [0;1], we can simply compute the integral of the VPI by sampling.

(12) $$E[G_{s,\;a}(x)]=\int_{0}^{1}G_{s,\;a}(x)P(Q_{s,\;a}^{\text{cache}}=x)\;dx$$

Instead of Boltzmann exploration, we employ ϵ-greedy exploration when choosing an action, i.e., at each decision point, a random action is uniformly sampled from the options with probability ϵ. This approach was chosen because given more complex tasks, the different learning speed of both subsystems may cause their *Q*-values to be of considerably different magnitude. In such cases, Boltzmann exploration is implausible, as it would virtually eliminate the chance of attempting an underestimated action, and thus prevent the system from learning its true value.

## 3. Results

Our model is evaluated in a number of settings, which can be divided into two broad classes. The first consists of variations of a simple feeder task, identical to those by Daw et al. (2005), which are to show that even with the modifications introduced here, the model still reproduces the basic devaluation resistance effects of its predecessors. Afterward, we will examine our central phenomena related to intrinsic motivation and activity in a more complex, “creative” task. Here, we take creative to mean a problem that requires a long chain of actions to solve, where each action does not cause a visible approach toward the goal.

### 3.1. Devaluation resistance

Daw et al. (2005) and Keramati et al. (2011) mostly examined their respective models using a decision task inspired by experiments with rats (Holland, 2004, Killcross and Coutureau, 2003), where the animals needed to manipulate a feeding apparatus in a short sequence to generate a reward. Those sequences had a maximum length of two decision points, and either two or three possible actions were available.

The first, simpler variant of the task allows the agent to choose between two actions, representing a lever press and a magazine entry. Only a press followed by an entry generates any reward, while any other sequence leads to a restart. In a second variation of moderate difficulty, there is an additional chain-pulling action, which, if followed by a magazine entry, leads to a different, but equivalent, extrinsic reward.

We perform the same series of experiments, with largely identical parametrization. Those that were changed, as well as newly introduced ones, are summarized in Table 1.

**Table 1:** **Default parameters that were used in the feeder task**.

**Parameter**	**Symbol**	**Value**
Search costs	τ	0.1
Exploration	ϵ	0.2
Intrinsic reward factor	ι	2.0

To examine the system's habituation, we devalue the goal state that is reached through the lever press by resetting its extrinsic reward distribution to Beta(1, 15). This is done after both moderate (20 episodes) and extensive training (200 episodes), and the changes in the ratio at which the lever is pressed is observed. In the moderately difficult setting, the devaluation takes place slightly later after 240 episodes to account for the more difficult task.

**Figure 3:**
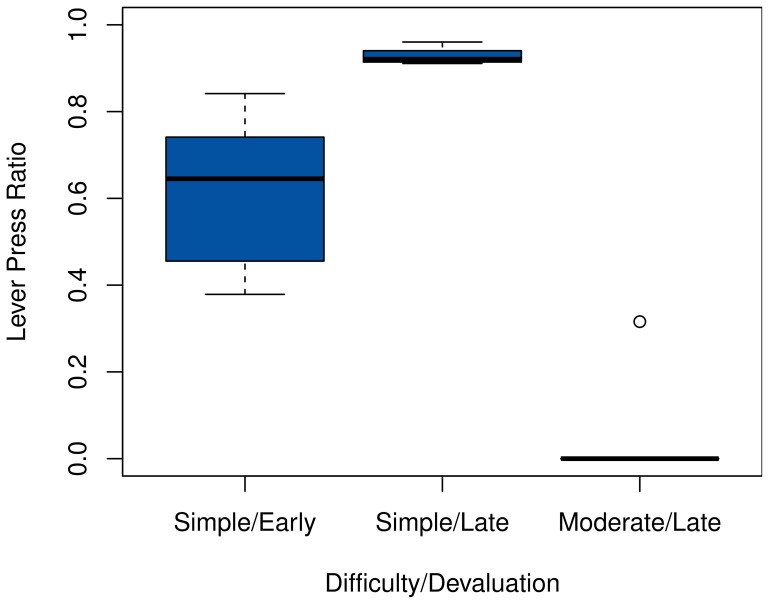
**Post-devaluation frequency of choosing the lever press action in the starting state**. Values are relative to their respective pre-devaluation rates. Comparisons are between the 100 cycles before and after the devaluation for the late settings, and 20 cycles for the early one. Note that action choices are determined before exploration, hence the sharp drop in the moderate task.

The results for all settings, summarized in Figure 3, are consistent with those of the predecessor models. While the system generally reacts more quickly to an early devaluation in the simple setting, its behavior does not change readily after extensive training, due to the inflexible habitual system having become active. The effect of early devaluations exhibits a much higher variance, which is to be expected; considering the random nature of exploration, the degree to which the system has learned the optimal policy and become habituated can differ considerably after a mere 30 episodes.

**Figure 4:**
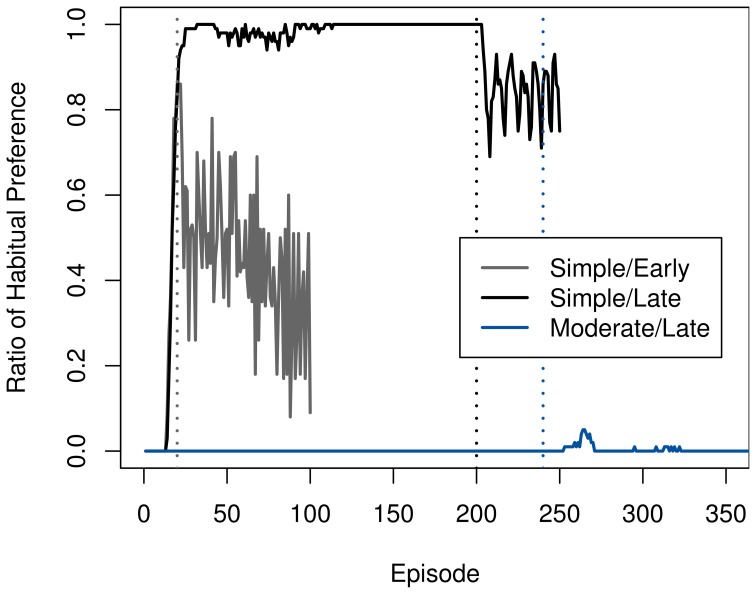
**Development of the ratio of selecting the habitual system over the goal-directed one in the starting state in the rat experiment**. Ratios in each time step were averaged across 100 runs. Devaluation points are marked using dashed lines in the matching color.

The speed of adaption mirrors the rate at which the goal-directed system was used around the time of devaluation, as Figure 4 illustrates. In the moderate task, the ambiguity of the two available actions causes a persistently high VPI and thus a continued use of the goal-directed system. Coupled with the high accuracy of the transition model after extended training, this allows the agent to switch to the chain-pulling action immediately.

### 3.2. Reward-based activity

The above feeding tasks consist only of very few states and actions, making them too simple to showcase those phenomena related specifically to intrinsic motivation. We therefore consider a more complex setting, adapted from the Playroom environment used by Singh et al. (2005), albeit simplified to accommodate the use of exact inference Bayesian RL.

In this task, the agent has to learn to manipulate a number of objects, each of which causes a different effect when used. A blue box can be used to start playing music, while a red one stops it. A switch toggles the lighting of the room, which causes the colored boxes to become indistinguishable. Lastly, there is a toy monkey, which does not cause any effect and serves as a neutral distractor. These objects need to be used in a specific sequence to bring about some desired goal state, which differs between experiments. Generally, the goal is to turn the music on and the light off, with additional success requirements in some settings.

The agent possesses a hand and an eye, both of which must rest on an object for it to become usable. Aside from performing an object affordance, the agent can also move its eye to a random object, bring the hand to the object the eye is resting on, or perform a null action that has no effect whatsoever. The null action generates a small positive action reward, unlike the other actions which cause negative ones. We thereby model an agent's general tendency to prefer the action that exerts the least effort.

While still simple for a task aimed at intrinsic motivation, it is considerably more complex than the food dispensal experiments. Most notably, trajectories can be cyclic, and one of the actions is non-deterministic. In addition, the partial observability of the state when the light is off can lead to local minima in the policy.

In this framework, we observe the behavior of the system using different combinations of intrinsic and extrinsic rewards, and determine whether the phenomena described in section 1 can be reproduced. Action rewards are present in all cases.

Unless noted differently, the system was parameterized as in Table 2. Most notably, the forgetting factor θ, the reward horizon η and the Dirichlet initialization α_*i*_ were adjusted to account for the longer episodes and more complex process model; otherwise, the system would forget old experiences faster than it could collect new ones. The action rewards *r*_*a*_ were always very slightly positive (0.005) for the null action, and negative (−0.02) for all others. They thereby model the intuitive assumption that if doing nothing promises the same reward as performing an action, the null action should be preferred. At the same time, the use of the null action should not accumulate too high action rewards, lest it overshadow those arising in the terminal states, where a terminal extrinsic reward of 1 was given.

**Table 2:** **Default parameters that were used in the Playroom task unless noted otherwise**.

**Parameter**	**Symbol**	**Value**
Forgetting factor	θ	0.9999
Search costs	τ	0.1
Update rate of avg. reward	η	0.001
Exploration	ϵ	0.2
Intrinsic reward factor	ι	2.0
Initial transition model	α_*i*_	0.1
Reward discount factor	γ	0.95

#### 3.2.1. Activity without extrinsic rewards

**Figure 5:**
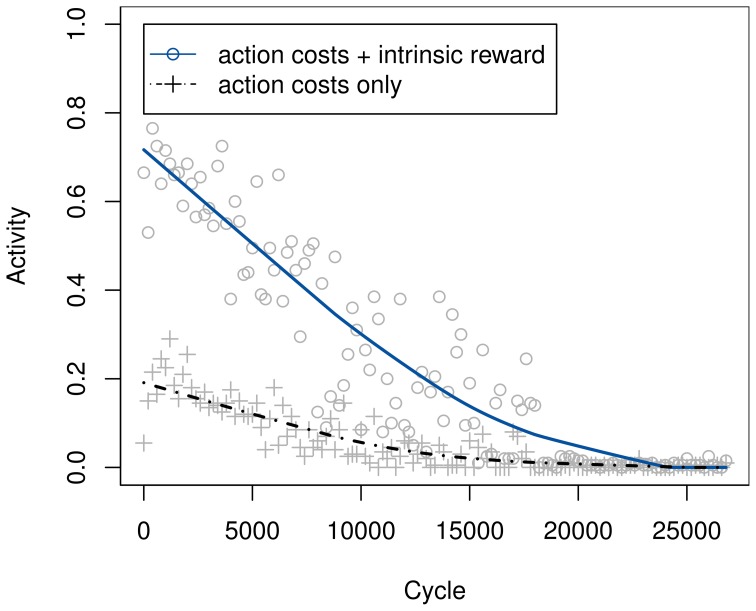
**Development of the percentage of non-null action choices, with and without intrinsic rewards**. Curves are based on the theoretical greedy choice of action, even in the 20% of cycles in which an ϵ-greedy exploratory action was ultimately used. Ratios were determined across bins of 200 samples and smoothed using locally weighted scatterplot smoothing.

A first experiment compares the activity of the system with and without intrinsic rewards. In this setting, there are no external rewards whatsoever, aside from the action-dependent transition costs. One would intuitively expect the overall activity, i.e., the occurrence of non-null actions, to be increased when using intrinsic rewards—higher motivation should naturally lead to more activity. And indeed, as Figure 5 illustrates, their use leads to a significantly lower rate at which the null action is chosen.

The activity with intrinsic motivation drops to a similar level as without it only after extensive training, once the model has stabilized and no more intrinsic reward can be generated. This effect seems plausible as well, seeing as how even a motivated individual will eventually cease playing or being otherwise active. It is caused by the retaining of action-dependent costs, which will always cause the system to settle on the null action in the end.

#### 3.2.2. Post-extinction activity

To show that stronger extrinsic rewards lead to less activity, as proposed in section 1.2, we next have the system learn a policy while providing the maximum extrinsic reward upon entering the goal state *s*_+_. In this case, *s*_+_ is reached by having the music turned on and the lights off. We devalue it either after 50 or after 200 episodes of training by replacing the distribution of the extrinsic reward model for the goal state with the Beta distribution Beta(1, 15). The parameters of the replacement distribution were chosen in accordance with Daw et al. (2005) in such a way as to concentrate most of the probability mass at 0. Note that we devalue the goal, rather than merely extinguishing its extrinsic reward, under the assumption that for higher-level intelligent agents, an extinction will be registered immediately, like a devaluation.

**Figure 6:**
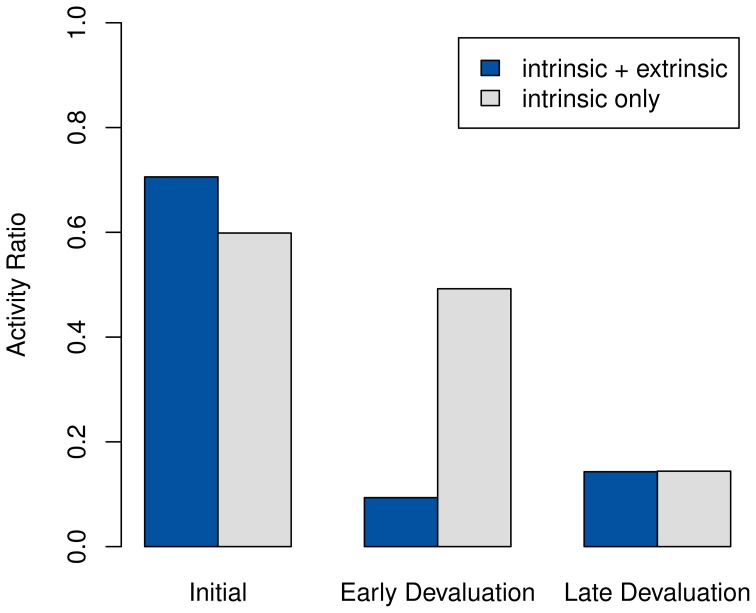
**Percentage of non-null actions chosen by the system using both intrinsic and extrinsic rewards, compared to activity using only intrinsic motivation**. The goal state is devalued after episodes 50 and 200.

If the devaluation occurs early, the post-devaluation activity drops sharply compared to its earlier level, as shown in Figure 6. In contrast, the purely intrinsic system remains active during the same time period. Only considerably later, once all intrinsic motivation in the model has been exhausted, does it become as inactive as the system using extrinsic rewards does after the devaluation.

**Figure 7:**
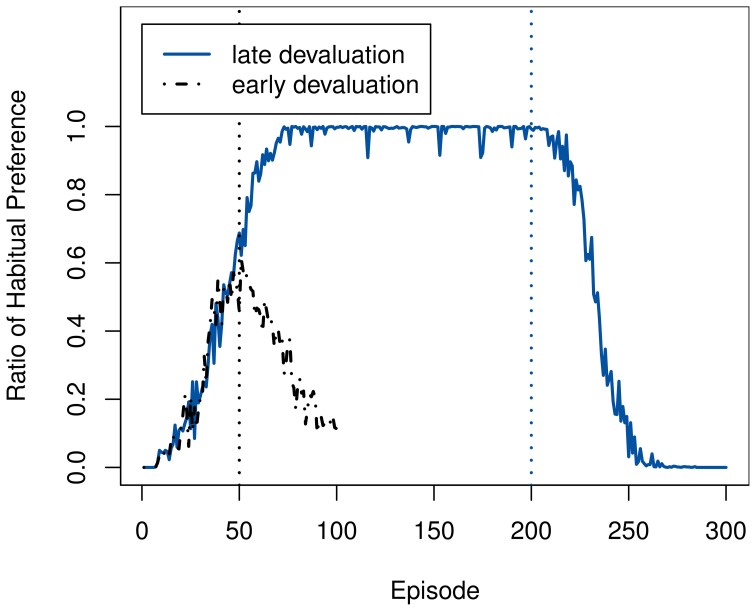
**Ratio of how often the habitual system is selected vs. the goal-directed one, when using both intrinsic and extrinsic rewards**. The vertical lines mark the times of devaluation at 50 and 200 episodes.

The lowered activity is in fact caused by the re-activation of the goal-directed system. As the costs of opportunity for performing a tree-search decrease, it takes over from the habitual system as seen in Figure 7. The previous takeover of the habitual system caused the agent to be active mostly in a limited region of the state space, as any exploration attempts were cut short by the habitual system's drive to reach the goal. Consequently, the model in this area of the state space is very accurate already. Therefore, no intrinsic reward is generated anymore, and the goal-directed system will not deviate from its path once having taken over. Essentially, due to the prolonged activation of the habitual system, the intrinsic motivation will have been exhausted without having the chance to cause any increased exploration and activity.

Also note that in Figure 6 the purely intrinsic setting results in slightly lower initial activity than the pre-devaluation case. This observation seems plausible, since a system not driven by extrinsic rewards would be more likely to try the sub-optimal null action to improve its model.

#### 3.2.3. Scope of motivation

One assumption we made was the local scope of intrinsic motivation. In accordance with Equation (8), the intrinsic reward *I* is only applied to the final *Q*^tree^ after the tree-search. Therefore, only the progress in the transition model out of the current state is considered when generating *I*.

One possible alternative would be to not consider intrinsic rewards locally, but globally, by applying them to the target mean already during tree-search. To do so, one would merely have to revise Equation (9) to

(13) $${\hat{\mu }}_{s,\;a}^{\text{tree}}=\mu_{s^{\prime },\;a_{*}}^{\text{tree}}+r_{a}+I_{s,\;a}$$

This should drive the agent more strongly into areas of the state space it has not observed yet, facilitating the acquisition of a better model.

**Figure 8:**
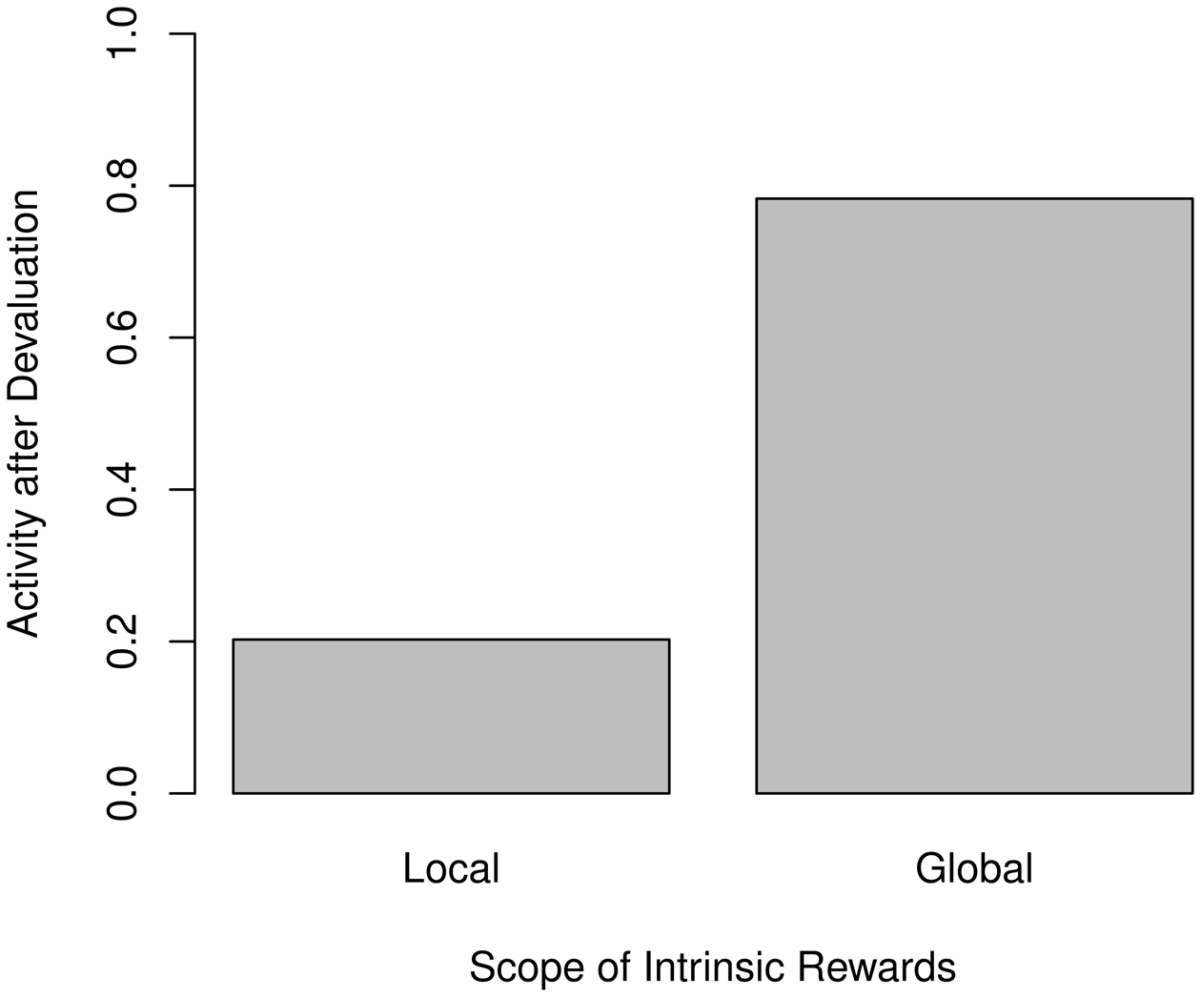
**Relative amounts of non-null action after devaluation for local (left) and global (right) intrinsic rewards**. Activities are normalized to the pre-devaluation level.

However, the assumption of global intrinsic rewards is inconsistent with the empirical findings. Figure 8 compares the post-devaluation activity between both approaches, and it becomes clearly apparent that the previously observed reduction in activity becomes much less pronounced when using global motivation.

### 3.3. System performance

While intrinsic rewards as modeled here account for the above activity phenomena, there have been no considerations of learning performance. Therefore, we also perform a number of experiments in the same setting as before to examine the overall performance of the system.

#### 3.3.1. Model acquisition

Clearly, a sensible model of intrinsic rewards should also justify their existence, as one would expect them to aid learning in some manner.

We thus test the system with and without intrinsic motivation in a task in which we change the goal state after a period of training. Initially, the agent receives an extrinsic reward for turning on the music and switching off the light as before, regardless of where the hand and eye are placed when the two conditions are met. After 200 episodes, one variation of the goal is devalued: if hand and eye are on a box when the music and light conditions have been met (i.e., if the light has been left off), no more extrinsic reward is given. The other possibility of having the hand and eye on the switch at the time, i.e., (turning the light on before manipulating the music, then turning it off again) remains as before. The first combination, which we will refer to as the *proximal goal*, can be potentially reached in as little as three steps, while the second *distal goal* requires three times as many.

**Figure 9:**
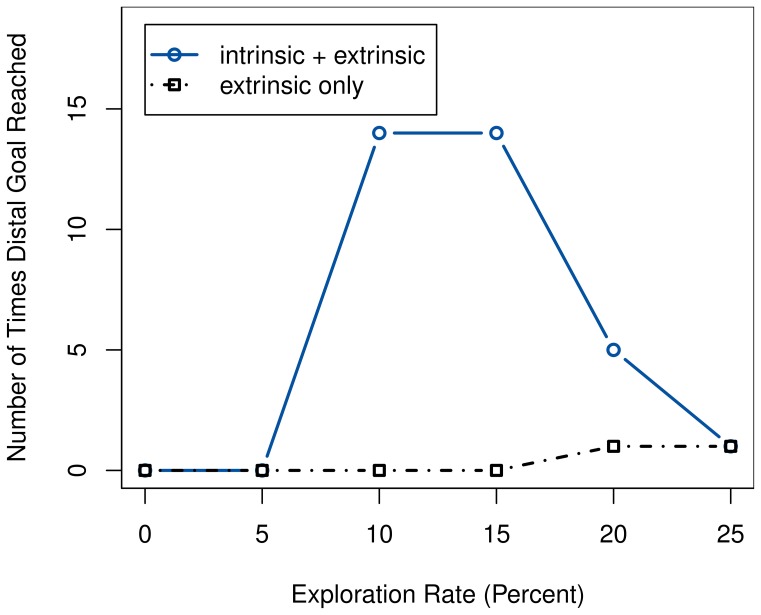
**Comparison of the number of times the agent reaches the distal goal after devaluation during 100 test episodes, with and without intrinsic rewards, for different exploration rates**.

The setup is repeated both using intrinsic rewards and using only extrinsic ones. We observe the frequency at which the agent manages to reach the remaining goal state after the devaluation. While it almost never enters the distal when only extrinsic rewards are given, it does manage to do so more often if using intrinsic motivation. The effect is not completely independent of the ϵ-greedy exploration; as figure Figure 9 illustrates, even the intrinsically motivated system fails to find the distal goal in case of too low a value for ϵ. Similarly, excessive over-exploration causes the performance to drop as well, as it prevents the agent from performing its learned policy. Regardless, the system clearly performs better with intrinsic reward than without, and this effect is even more pronounced if using slightly lower values for ϵ.

The results can be explained by the intrinsically motivated system's drive to better explore the state space. Thus, it possesses a higher change of finding the distal goal state. Ideally, the agent should then directly learn to prefer the distal route, as it provides a guaranteed extrinsic reward—unlike the proximal one, due to the inability to differentiate the box colors while the light is off. But even if it does not, having seen this alternate goal would enable it to immediately switch over to it once the devaluation occurs.

#### 3.3.2. Effects of promised rewards

As a final experiment, we examine how the model accounts for phenomena related to promises of extrinsic rewards. As observed by Ariely et al. (2009), a high expectation of being rewarded later upon completion of a task can actually reduce an agent's performance in complex tasks compared to a purely intrinsically motivated individual.

A reasonable assumption to simulate promises of later rewards seems to be to fix the average reward *R* at 1, i.e., treat the promise of extrinsic rewards just the same as their observation. We thus take *R* as the *expected* reward, rather than the *observed* average. In fact, this assumption is closer to those of Niv et al. (2007), whose model of tonic dopamine levels Keramati et al. (2011) have based the concept of *R* on.

The conditions with and without fixed *R* are compared with respect to both the time spent on reasoning processes and the ability to learn a task. We also test in two different settings of distinct difficulty, both of which require the agent to turn the music on and the light off while looking at the blue box. In the *distal* task, the agent starts in the same configuration as before, with light and music off, while in the *proximal* setting, the music is already playing and the light is on, requiring it to perform a much simpler sequence of actions.

**Figure 10:**
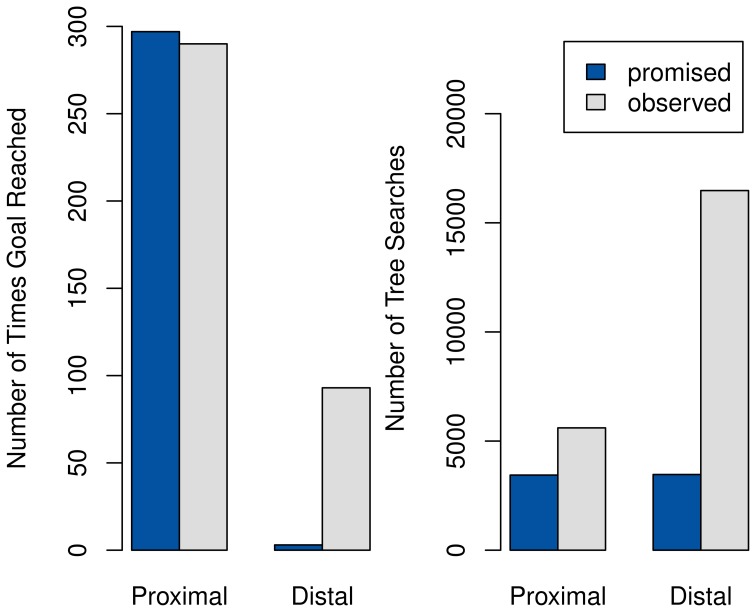
**Comparison of performance (left) and speed (right) between systems with promised extrinsic rewards (dark gray) and with only observed rewards (light gray)**. As one tree-search takes an average of 350 ms, compared to less than a millisecond needed for querying the habitual system, the number of tree-searches is directly proportional to the total time.

The results for 300 episodes of training are summarized in Figure 10. Using promises of extrinsic reward reduces the amount of time spent on tree-searches significantly, particularly in the distal setting. However, the speed improvement also comes at a drastic decrease in performance in complex tasks, with the fixed *R* completely preventing the agent from solving the distal case.

It should be noted that this behavior does not result from our additions to the model, but would already have been present in that of Keramati et al. (2011) using the slightly changed interpretation of *R* adopted here. It is included here mostly because it has been ignored in the prior work, despite its noteworthy consistency with the empirical findings of Ariely et al. (2009).

## 4. Discussion

We have proposed an extension to two previous models of the striatal learning system that introduces the concept of intrinsic motivation. By assigning additional intrinsic rewards for higher learning progress, we were able to reproduce several additional empirical phenomena that were not covered by our predecessors. In particular, we account for the fact that the presence of intrinsic motivation predictably raises the overall activity, but that it can be suppressed by high extrinsic rewards in turn. We have also shown that intrinsic rewards lead to better system performance in more complex tasks requiring creative solutions.

Of course, there are always aspects of the model that could be improved or require clarification, as well as behaviors that have not been examined empirically yet. These will be discussed in more detail in the following.

### 4.1. Computational intrinsic motivation and related biological models

The principles of intrinsically motivated learning have gained increasing interest in the field of computational RL. Formalization of different aspects of intrinsic motivation, such as curiosity or competence, are expected to provide general, task-independent mechanisms that let artificial agents explore their own skills and their environment efficiently and autonomously. Furthermore, the models, which the agents build through environment interaction guided by intrinsic motivations, promise to enable improved adaptability to environmental changes or new task requirements.

Starting with the pioneering work of Schmidhuber (1991a,b), who introduced curious model-building controllers that got rewarded strongest for (near-mismatches of) predictions about the world, to Singh et al. (2005) who used internal reward signals proportional to the agent's error in predicting salient events in a related way, many approaches that tried to formalize notions of interestingness, curiosity, competence, and improvement of an agent's model about the world have been proposed [e.g., by Oudeyer et al. (2007), Schembri et al. (2007), Schmidhuber (2008), Baranes and Oudeyer (2009), and Grzyb et al. (2011)]. Here, our focus is on computational mechanisms that could explain phenomena observed in the psychology literature, i.e., on cognitive modeling, rather than proposing general-purpose reward mechanisms. A full overview of approaches from the computational RL literature is therefore beyond the scope of this article. Surveys for this purpose, however, such as Oudeyer and Kaplan (2007) and Schmidhuber (2010), as well as the recent book by Baldassarre and Mirolli (2013) give a much more complete picture in this regard.

Recent models dealing with aspects of intrinsic motivation from a biological perspective include those in Bolado-Gomez and Gurney (2013) and Mirolli et al. (2013). Both of these propose a role for the phasic dopamine signal from dopaminergic neurons in the brain, and both strive for consistency with neuroscientific data. In Bolado-Gomez and Gurney (2013) the authors suggest that this signal indicates surprising actions outcomes, and that objects associated with such outcomes acquire a novelty salience. They show that these signals can be used by an agent for the purpose of action discovery. In Mirolli et al. (2013), on the other hand, it is proposed that phasic dopamine signals reward prediction errors which are shaped by two different kinds of reinforcers: temporary, internal rewards for unexpected stimuli the agent experiences, and permanent, external rewards of a biological nature. Based on this assumption, phasic dopamine can drive both discovery and learning of new actions in a unified way. The model we present here also relies on an internal reinforcer which the agent can perceive in case its model of the world changes (see below). However, at this point, we do not identify the exact source of this signal. In future studies, it might be interesting to examine whether our proposed mechanism would fit the empirical data about phasic dopamine release though.

#### 4.1.1. Alternative mechanisms of motivation

The underlying assumption behind our concept of intrinsic motivation is higher learning progress yields increased rewards. To measure progress, we observed shifts in the model's distribution means. This approach is inferior to tracking reductions in the distribution variance, as it does not allow us to differentiate between actual learning progress and cases where the model simply cannot be learned, for instance due to non-determinism. However, as described in section 2.2.1, the variance cannot be used when using Dirichlet distributions. Therefore, future improvements should try to either replace the distribution type used, or examine if alternate types of intrinsic motivation still exhibit the same behavioral effects.

#### 4.1.2. Applicability to larger problems

In this work, we were focused purely on the explanation of empirical phenomena. For the sake of a clean theory, we used exact inference Bayesian RL. However, this approach quickly becomes intractable when applied to more complex problems. Both from a pragmatic standpoint as well as from a theoretical one—after all, rats and humans are capable of solving problems more difficult than pressing a lever or manipulating a small number of objects in sequence—it would therefore be desirable to replace it with approximative methods. Ideally, the observed phenomena should remain in that case. The ability to solve more complex tasks would also enable us to truly examine the validity of the model and of different hypotheses of motivation quantitatively.

#### 4.1.3. Isolated treatment of actions

In our model, just like in those of our predecessors, we assume that the choice between the habitual and the goal-directed system is made independently for each available action, and only afterward exploration is performed over the resulting *Q*-values. Therefore, once the VPI approaches the threshold *R*τ, the habitual system may take over for single actions, but not for others. This can potentially lead to sub-optimal behavior if both systems learn at different speeds, as is often the case for complex tasks. If, then, the goal-directed system has a lower estimate than the habitual one, its prediction will be disregarded during exploration, despite generally being more accurate.

While this effect does not usually prevent learning, as either the sub-optimal action will also drop to its true level over time, or its VPI will decrease below threshold, this may reduce the speed at which the system learns to solve a task. Thus, for practical applications, one might either use the goal-directed system to determine all actions' values if even one calls for it, or re-calculate the VPI for all actions immediately after performing a tree-search. These approaches should still account for all observed phenomena, and may be worth examining in future works.

#### 4.1.4. Integration with other models

A model for the division of the decision-making system in rats has also been proposed by Caluwaerts et al. (2012), albeit with the goal of explaining a different type of behavior entirely, namely navigation. While their design of a learning arbitration mechanism does not readily afford a speed/accuracy trade-off as introduced by Keramati et al. (2011), their use of learning progress to detect context changes (i.e., a shift in the goal state) could prove compatible with our model and potentially be employed to replace the explicit devaluation used so far.

### 4.2. Parametrization

The model was generally designed to be robust to the choice of its parameters. Usually, their exact values should only affect the speed at which the system learns and the time at which the observed phenomena occur. However, there are a few parameters that influence the principal behavior of the system.

#### 4.2.1. Search costs

In our model, we adopted the VPI-based competition mechanism of Keramati et al. (2011) for its high plausibility and larger compatibility with intrinsic rewards. It should, however, be noted that the choice of the active subsystem in Equation (5) depends heavily on the search costs τ. Since the habitual system's value distributions may always overlap to some extent, the VPI will generally converge to some non-zero value. Thus, if τ is chosen too small, the habitual system may never become active as the tree-search can be performed practically for free. Conversely, too high a search cost will prevent the goal-directed system from being chosen. The fact that the same setting of τ = 0.1 can be used both for the simple feeder task and the more complex Playroom suggests that the admissible range of τ is wide enough to not require an exhaustive search. Even so, in principle it may be necessary to choose τ appropriately in different tasks.

#### 4.2.2. Forgetting factor

One aspect that the system is fairly dependent on is the forgetting factor θ. With θ = 0.98, as used by Daw et al. (2005), it is impossible to learn a task as complex as the Playroom setting, since the distribution parameters will usually decay back to their priors faster than new experiences are acquired. This requires us to tune the parameter closely to the task complexity.

In this work, we settled for a setting of θ = 0.9999, therefore practically turning parameter decay off. This approach comes at a cost, in turn, as it makes it difficult to change the system's behavior after a while. Once the distributions have stabilized after extensive training, new experiences will be virtually ignored. Also, when learning tasks with a larger state space, the acquisition of the Dirichlet transition model may take noticeably longer than learning a policy along a narrow trajectory in the habitual system, causing the latter to become severely over-trained.

While such behavior can actually be realistic—after all, a habit usually takes very long to unlearn—it would effectively render the habitual system useless in real-world applications. For its existence to be truly plausible, the system needs to be extended with a more robust mechanism for forgetting experiences. One option would be a surprise-based approach, which causes the parameter decay to accelerate when an unexpected event occurs, while gradually slowing down otherwise.

### 4.3. Predictions

Our model makes a number of assumptions and shows behaviors that have not been examined in empirical studies to date. These predictions could therefore be used to support or falsify the model.

#### 4.3.1. Scope of motivation

In section 3.2.3 we found that in order to reproduce the empirical effects on activity, we have to assume that intrinsic motivation is local in scope rather than propagating all the way through the model. To our knowledge, no studies regarding the scope of intrinsic rewards exist, and it remains unclear how one could test such an aspect empirically. A possible approach could be to have individuals perform a creative task before introducing an unexpected event into the process. In two conditions to be compared, this event should either be immediately reproducible by the subject, say by pressing a previously unavailable button, or require a long sequence of actions to bring about. By observing whether the longer sequence causes less exploration in its direction or not, it should be possible to confirm or falsify our locality assumption.

#### 4.3.2. Promised rewards

In section 3.3.2, we adopted the hypothesis of Niv et al. (2007) that expected rewards are explicitly encoded in the striatal system through tonic dopamine levels. In the framework of our model, assuming that the average reward *R* encodes expectations leads to a system behavior that matches empirical findings by Ariely et al. (2009). Our model therefore supports the prediction that promises of rewards should indeed increase dopamine levels. However, the dopamine level theory is untested thus far, and would require an empirical study to confirm. Furthermore, a more detailed examination how promises of varying degrees influence behavior would be in order.

### Conflict of interest statement

The authors declare that the research was conducted in the absence of any commercial or financial relationships that could be construed as a potential conflict of interest.
